# Deficits of facial emotion recognition and visual information processing in adult patients with classical galactosemia

**DOI:** 10.1186/s13023-019-0999-3

**Published:** 2019-02-26

**Authors:** Mirjam Korner, Sonja Kälin, Antoinette Zweifel-Zehnder, Niklaus Fankhauser, Jean-Marc Nuoffer, Matthias Gautschi

**Affiliations:** 1 Institute of Clinical Chemistry, University Hospital Bern, Inselspital, Bern, Switzerland; 2 Division of Paediatric Endocrinology, Diabetology and Metabolism, Department of Paediatrics, University Hospital Bern, Inselspital, Freiburgstrasse 15, CH-3010 Bern, Switzerland; 3 Division of Neuropaediatrics and Neuropsychology, Department of Paediatrics, University Hospital Bern, Inselspital, Bern, Switzerland; 4 Present address: Division of Neurology and Neuropaediatrics, Department of Internal Medicine and Paediatrics, Kantonsspital Graubünden, Chur, Switzerland; 5 Statistics, Clinical Trial Unit, University of Bern, Bern, Switzerland

**Keywords:** Classical galactosemia, CANTAB, Facial emotion recognition, Visual information processing, Neuropsychology, Executive function, Sustained attention

## Abstract

**Background:**

Classical galactosemia (CG) is due to a severe deficiency of the galactose-1-phosphate uridyl-transferase (GALT), the main enzyme of galactose metabolism. Even early introduction of galactose-restricted diet fails to prevent long-term complications, including cognitive impairment, neurological and psychiatric problems, osteoporosis, premature ovarian failure and infertility. Detailed neuropsychological phenotyping is needed in order to better understand the relevant neurodevelopmental deficiencies and to develop effective treatment strategies.

**Aim:**

To define specifically and significantly impaired neuropsychological traits in adult CG patients of the Swiss cohort.

**Methods:**

Prospective cohort study. 22 CG patients, with confirmed genotype and low GALT activity, and 15 controls completed a computer-based neuropsychological test battery (CANTAB). Additionally, broad IQ evaluation was made for the CG patients.

**Results:**

In most outcome measures of the CANTAB tasks, CG patients performed significantly worse than controls. The deficits in CG patients were most prominent in tasks that involve rapid visual information processing and facial emotion recognition.

**Conclusion:**

CG patients have specific cognitive problems such as impaired visual information processing and facial emotion recognition. The deficits in facial emotion recognition have not been described before and could help explain difficulties in social interactions often experienced by patients with CG.

**Electronic supplementary material:**

The online version of this article (10.1186/s13023-019-0999-3) contains supplementary material, which is available to authorized users.

## Background

Classical galactosemia (CG; OMIM #230400) is a rare disorder affecting the galactose metabolism. It is autosomal-recessively inherited and caused by a profound deficiency of the enzyme galactose-1-phosphate uridyltransferase (GALT; EC 2.7.7.12) [1]. Together with two other enzymes: galaktokinase (GALK) and UDP-galactose epimerase (GALE), GALT is part of the Leloir pathway that metabolizes ingested galactose into glucose-1-phosphate used for energy and into UDP-galactose which is used for glycosylation of complex molecules. In untreated CG patients, galactose, galactose-1-phosphate, galactitol and galactonate accumulate in body tissues and fluids [2]. So far over 180 different mutations in the gene encoding for GALT have been identified and associated with CG [3]. Other mutations are known to cause only mild deficiency, including the so-called Duarte-2 mutation (N314D), the most common of them. Compound-heterozygotes for a Duarte and a classical mutation normally have a residual GALT activity of 14–25% and a good prognosis without treatment [4].

Newborns with CG develop a life-threatening intoxication syndrome with acute liver failure, renal tubular dysfunction, sepsis and cerebral oedema. Symptoms resolve within a few days after establishing a galactose-restricted diet [1, 2]. Even when typical clinical signs of galactosemia are present, the diagnosis may be missed. In order to ensure rapid diagnosis and adequate management, newborns are screened for galactosemia in many countries. Newborn screening can decrease the morbidity and mortality caused by the acute complications of galactosemia in the neonatal period.

However, even strict adherence to the diet cannot prevent the long-term complications that may occur in CG, such as deficits in cognitive functions, speech and language impairments and neurological deficits including tremor and other extrapyramidal motor abnormalities, as well as premature ovarian insufficiency and low bone mineral density [1, 5, 6]. Most studies researching the neuropsychological impairments have focused on global measures of IQ, reporting overall IQ scores within the low to below average range, with a great variability between individual patients [7–14]. The cognitive impairments are thought to result from a broad set of deficits [10]. Few other studies also described visual-perceptual difficulties and less well-developed executive functions [7, 12, 15]. Many of the patients suffer from speech and language impairments [16, 17]. Higher incidence of psychiatric disorders such as depression or anxiety and problems with social interactions are also known in CG [17]. The biomarker galactose-1-phosphate, correlates only loosely with long-term neuropsychological and motor outcome [5, 18, 19].

The exact pathomechanisms of these neuropsychological long-term impairments remain unclear. The brain may already be damaged in utero, as elevated levels of galactose-metabolites were found in foetuses from the age of 20 weeks of gestation [20]. Others suggested that the endogenous galactose production might lead to a toxic accumulation of galactose metabolites even when patients are on a galactose-restricted diet [1]. The currently prevalent theory is that abnormal galactosylation of complex molecules, including myelin, may contribute to the pathology [21, 22]. Neuroimaging studies showed poor myelination and other white matter abnormalities, as well as cerebral and cerebellar atrophy [10, 23, 24].

In order to further characterize the neuropsychological profile of adult patients with CG, especially aspects of executive and visual perceptual functions, we used selected tasks from the Cambridge Neuropsychological Test Automated Battery (CANTAB), a computerized assessment tool. This battery is highly standardised and easy to conduct, and the results highly reproducible.

## Results

### Characteristics of patients, Duarte subjects and controls (Table 1)

Twenty-two patients, i.e. 58% of the known Swiss CG population ≥ 16 years of age, were enrolled in the study. Thirteen (59%) were females. Mean age was 30 years (*SD* 11), median 29, range 16–59 years. Of the 22 CG patients all except one were diagnosed through newborn screening. Most of them nevertheless exhibited symptoms of intoxication before treatment initiation. All patients had two known classical mutations (Q188R, K285 N, L195P, H319Q, A320T and M142K), except two patients with one classical and one known slightly milder (R258C) mutations and one patient with the genotype Q188R/L264 V and residual GALT activity of 1.5%. Mean IQ score of the CG patients was 77 (*SD* 17), the median also 77, and the range 49 to 112. Comparison of patient and control groups did not show significant differences concerning age (*p* = 0.475) and gender (*p* = 0.903). The education levels of controls and the parents of the patients were comparable (*p* = 0.383). But, as expected, they differed significantly between patients and controls (*p* = 0.028). The three subjects with mild “Duarte” galactosemia were compound-heterozygous for a classical and the Duarte-2 (N314D) mutations. They were clinically normal.

**Table 1:** Characteristics of Patients, Duarte and Controls

	Duarte	Controls	Patients	*p*-value
*n*	3	15	22	
Age: Mean (*SD*)	33.3 (3.2)	33.1 (11.7)	30.4 (11.1)	0.475
Median (range)	34.0 (32–37)	32.0 (21–61)	28.5 (16–60)	0.467
Gender = Female (%)	2 (66.7)	10 (66.7)	13 (59.1)	0.903
IQ: Mean (*SD*)	115 (15)	-	77 (17)	**0.028**
Median (range)	108 (105–133)	-	77 (40–112)	**0.011**
Education subjects (%)				
• School without qualification	0 (0.0)	0 (0.0)	3 (13.6)	0.380
• School with qualification	0 (0.0)	0 (0.0)	2 (9.1)	0.645
• Vocational	0 (0.0)	9 (60.0)	15 (68.2)	0.872
• Undergraduate	1 (33.3)	1 (6.7)	2 (9.1)	1.000
• Postgraduate	2 (66.7)	5 (33.3)	0 (0.0)	**0.015**
Max. Education parents/ education controls (%)				0.383
• Vocational	0 (0.0)	9 (60.0)	11 (52.4)	0.792
• Undergraduate	2 (66.7)	1 (6.7)	5 (23.8)	0.397
• Postgraduate	1 (33.3)	5 (33.3)	5 (23.8)	0.737

*SD* standard deviation. *p*-values refer to comparison of patients with controls, significant values are in bold. The Student’s t-test was used for mean comparison and the Wilcoxon rank-sum test for median comparison. Chi-squared test was used for categorical variables. See methods section for description of the levels of education

### Validation of controls

Mean Z-scores of controls were compared to the CANTAB norms by one sample t-test. Overall, the control group was comparable to the normative data cohort of CANTAB (see Additional file 1: Table S1). We therefore compared the test results of CG patients with the results of our control group.

### CANTAB testing

**Fig. 1:**
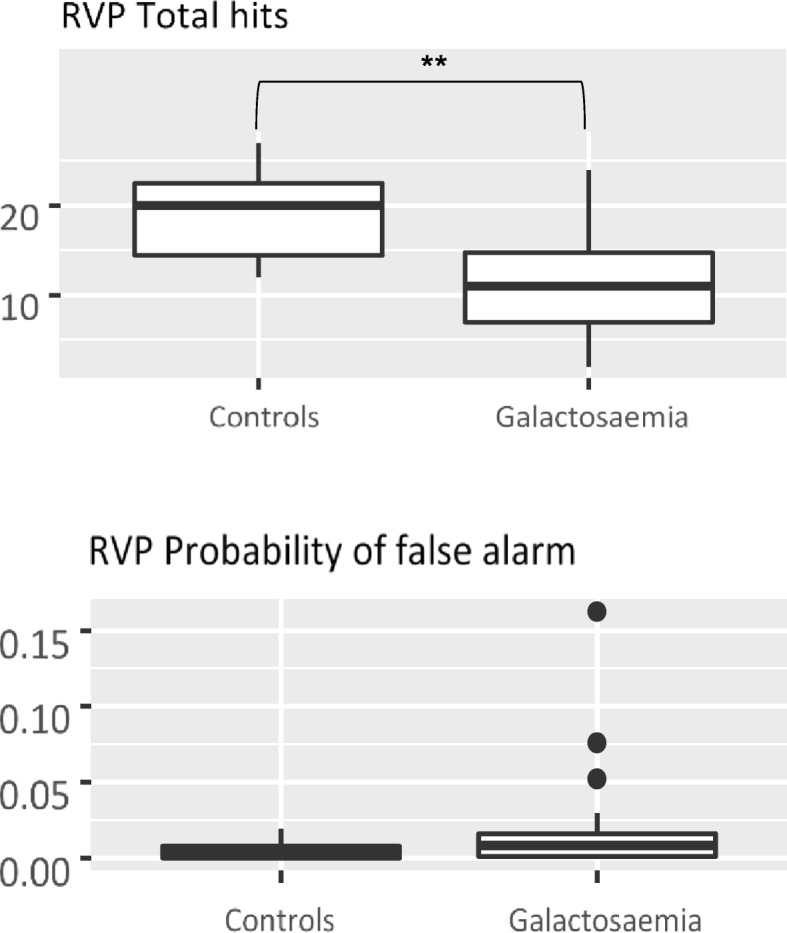
Rapid visual information processing (RVP) is a measure of sustained attention. Subjects had to recognise target sequences of three digits from numbers appearing in a pseudo-random sequence at a rate of 100 digits per minute. The number of total hits is significantly lower (** = *p* < 0.01) in Galactosemia patients compared to controls, whereas the probability of false alarms is not different in both groups

**Fig. 2:**
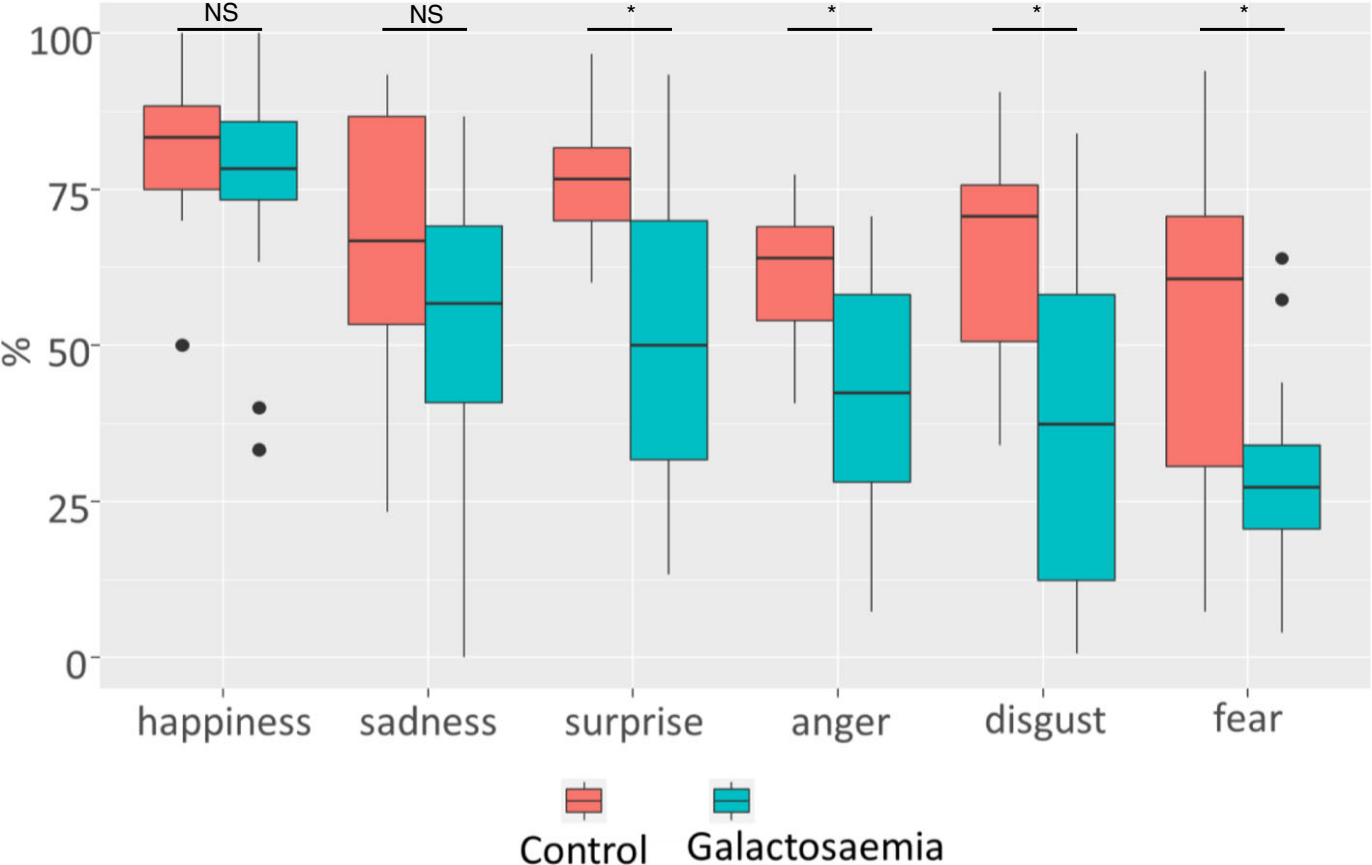
Emotion Recognition Task (ERT). The participants had to recognise facial expressions of six different emotions presented for 200 ms. Recognition of basic emotions, such as happiness and sadness, was not significantly different between patients and controls, whereas emotions considered more complex, including surprise, anger, disgust and fear, appeared significantly more difficult for patients than for controls. Percentage of correct recognition of each of the six emotions listed. NS = not significant, * = *p* < 0.05

Table 2 presents the descriptive data for all measures of the CANTAB. In the Motor Screening Task (MST), CG patients did not show a significantly longer mean latency or made more errors than controls. In the Paired Associates Learning (PAL) task, CG patients performed worse compared to controls. They needed more trials and made more errors until successful completion of a stage. Patients also made more errors in the stage with six shapes and needed more trials in total. However, none of these measures reached significance in nonparametric testing. In the Spatial Span (SSP) task, CG patients had a significantly shorter span length, which means they could remember shorter sequences than controls. Patients also made significantly more usage errors. These errors are made when the subject selected a box that did not change colour. In the Reaction Time (RTI) task, CG patients performed equally well as controls in the simple-choice part. In the five-choice part movement time was slower for CG patients compared to controls, again without reaching significance, because of the important variation and large overlap with controls. In the Rapid Visual Information Processing (RVP) task, CG patients performed significantly worse in all but one outcome measure. They were less likely to identify the target sequence but did not have more false alarms than controls (Fig. 1). The results of the Emotion Recognition Task (ERT) are displayed in Fig. 2: Recognition of the emotions happiness and sadness was not significantly different between CG patients and controls. However, CG patients had significantly lower percentages for the recognition of the emotions anger, disgust, fear and surprise. They also needed more time to answer than controls.

**Table 2:** CANTAB results

Test	Duarte		Control		Patients			
	Mean (SD)	*Median/range*	Mean (SD)	*Median/range*	Mean (SD)	*p*-value	*Median/range*	*p*-value
Motor Screening Task (MOT)								
1. Mean latency (ms)	628.5 (54.7)	*647/567–672*	803.8 (236.3)	*727/524–1221*	993.4 (284.0)	0.263	*942/593–1560*	0.150
2. Mean error	8.6 (4.6)	*8.0/4.3–13.5*	6.6 (1.9)	*6.0/3.7–10.9*	7.7 (2.1)	0.761	*7.4/5.1–12.2*	0.559
Paired Associates Learning (PAL)								
1. First trial memory score	21.3 (6.4)	*24/14–26*	20.7 (3.1)	*20/16–25*	18.6 (4.4)	0.200	*19/8–24*	0.778
2. Total errors adjusted	6.3 (9.3)	*2/0–17*	7.4 (5.9)	*6/2–22*	23.0 (31.3)	0.145	*16/2–148*	0.105
3. Total errors 6 shapes	3.0 (5.3)	*0/0–9*	2.5 (2.7)	*2/0–9*	8.8 (11.5)	**0.049**	*6/1–50*	0.321
4. Mean errors to success	0.79 (1.16)	*0.25/0.00–2.13*	0.92 (0.73)	*0.75/0.25–2.75*	2.25 (1.84)	**0.010**	*2.00/0.25–3.25*	0.105
5. Mean trials to success	1.25 (0.33)	*1.13/1.00–1.63*	1.36 (0.25)	*1.25/1.13–2.00*	1.79 (0.59)	**0.010**	*1.63/1.13–3.20*	0.103
6. Stages completed at first trial	7.0 (1.0)	*7/6–8*	6.1 (0.8)	*6/5–7*	5.7 (0.9)	0.263	*6/4–7*	0.778
7. Total trials adjusted	10.0 (2.6)	*9/8–13*	10.9 (2.0)	*10/9–16*	14.8 (6.4)	**0.041**	*13/9–37*	0.103
Spatial Span (SSP)								
1. Span length	7.3 (0.6)	*7/7–8*	7.1 (1.2)	*7/5–9*	5.6 (1.3)	**0.002**	*5.5/3–9*	**0.019**
2. Relative errors	1.9 (0.4)	*1.9/1.8–2.7*	2.2 (0.7)	*2.4/0.9–3.1*	2.2 (0.7)	1.000	*2.2/1.0–4.7*	1.000
3. Relative usage errors	0.14 (0.01)	*0.14/0.14–0.17*	0.32 (020)	*0.29/0.00–0.67*	0.60 (0.39)	**0.043**	*0.5/0.0–1.5*	0.556
Reaction Time (RTI)								
1. Mean simple reaction time (ms)	310 (63)	*295/255–379*	316 (57)	*300/254–459*	360 (94)	0.320	*335/266–594*	0.623
2. Mean simple movement time (ms)	337 (89)	*355/240–415*	403 (141)	*376/254–757*	472 (160)	0.320	*426/292–955*	0.568
3. Mean five-choice reaction time (ms)	338 (65)	*329/278–406*	337 (60)	*331/260–461*	393 (123)	0.284	*339/302–757*	0.437
4. Mean five-choice movement time (ms)	322 (64)	*336/252–377*	373 (87)	*358/254–567*	475 (151)	**0.031**	*420/291–827*	0.253
Rapid Visual Information Processing (RVP)								
1. A’ score	0.946 (0.055)	*0.962/0.884–0.991*	0.927 (0.045)	*0.930/0.858–1.000*	0.835 (0.078)	**0.002**	*0.837/0.677–0.972*	**0.009**
2. Probability of hit	0.790 (0.211)	*0.852/0.556–0.963*	0.719 (0.178)	*0.741/0.444–1.000*	0.426 (0.220)	**0.002**	*0.407/0.074–0.889*	**0.009**
3. Mean latency (ms)	382 (54)	*397/321–426*	408 (101)	*394/295–691*	563 (179)	**0.005**	*541/332–1070*	**0.019**
4. Probability of false alarm	0.004 (0.004)	*0.003/0.000–0.008*	0.005 (0.006)	*0.004/0.000–0.019*	0.020 (0.037)	0.409	*0.008/0.000–0.162*	0.434
5. Total correct rejections	261 (12)	*264/247–271*	255 (10)	*255/241–273*	235 (17)	**0.002**	*234/191–265*	**0.009**
6. Total hits	21.3 (5.7)	*23/15–26*	19.4 (4.8)	*20/12–27*	11.5 (6.0)	**0.002**	*11/2–24*	**0.009**
Emotion Recognition Task (ERT)								
1. Total number correct	134.3 (4.6)	*137/129–137*	120.0 (20.5)	*124/81–143*	85.8 (25.0)	**< 0.001**	*89/42–128*	**0.009**
2. Percent correct	74.6 (2.6)	*76.1/71.7–76.1*	66.7 (11.4)	*68.9/45.0–83.9*	47.7 (13.9)	**< 0.001**	*49.4/23.3–71.1*	**0.009**
3. Percent correct (happiness shown)	88.9 (11.7)	*90.0/76.7–100*	80.9 (12.2)	*83.3/50.0–93.3*	76.1 (15.4)	0.701	*78.3/33.3–100*	1.000
4. Percent correct (sadness shown)	80.0 (6.7)	*80.0/73.3–86.7*	66.9 (22.1)	*66.7/23.3–93.3*	51.8 (23.1)	0.145	*56.7/0.0–86.7*	0.360
5. Percent correct (anger shown)	73.3 (8.8)	*76.7/76.7–80.0*	60.7 (10.3)	*63.3/40.0–73.3*	41.1 (18.0)	**0.004**	*41.7/6.7–70.0*	**0.016**
6. Percent correct (disgust shown)	73.3 (12.0)	*70.0/63.3–86.7*	62.4 (18.0)	*70.0/33.3–90.0*	37.7 (26.0)	**0.009**	*36.7/0.0–83.3*	**0.048**
7. Percent correct (fear shown)	63.3 (15.3)	*66.7/46.7–76.7*	53.8 (26.8)	*60.0/6.7–93.3*	28.2 (14.6)	**0.005**	*26.7/3.3–63.3*	**0.048**
8. Percent correct (surprise shown)	68.9 (7.7)	*73.3/60.0–73.3*	75.3 (9.6)	*76.7/66.7–96.7*	51.1 (22.6)	**0.006**	*50.0/13.3–93.3*	**0.016**
9. Mean latency (ms)	1283 (447)	*1210/875–1761*	1616 (587)	*1379/988–2988*	2489 (922)	**0.018**	*2275/1320–4437*	**0.019**

CANTAB results from the six selected tasks with corresponding measures, expressed as means and standard deviation (*SD*), as well as medians and ranges of Duarte subjects, controls and patients. *p*-values refer to comparisons between patients and controls. The Student’s t-test was used for mean comparison and the Wilcoxon rank-sum test for median comparison. An adjusted *p*-value (FDR) was used throughout to account for multiple comparisons (see Methods). Significant values (*p* < 0.05) are in bold

### Importance of outcome measures

A random forest model consisting of 5000 trees was trained and validated on the CANTAB data to quantify the relative importance of each outcome measure for the discrimination of group membership (control vs. galactosemia patients; Additional file 2: Figure S1). The higher the MDA (mean decrease in accuracy) of a given measure the more important its contribution to group discrimination. Several global and individual outcome measures of the ERT (including total number correct, mean latency, as well as the emotions surprise and fear) and RVP tasks were most important for group discrimination. Bootstrap estimate of error rate: 35.14%.

### Correlations

A priori significant correlations of ERT and RVP outcome measures are shown in Table 3. When corrected for multiple comparisons (*p*-value FDR), only three measures, all from the ERT (total correct, recognition of *sadness* and *disgust*), correlated significantly with the overall IQ score of the patients but none with their level of education. In contrast, no significant a priori correlation at all was observed with the education of controls (not shown) or the maximum education of the patients’ parents. Note, that no correlation with IQ, educational level or age was found for the ERT *fear*, which was also important for group discrimination (see above). Two outcome measures, PAL total errors at the stage of six shapes and RTI five-choice reaction time showed a significant correlation with the age of the controls, but not with the age of patients (not shown). No significant correlations between CANTAB outcome measures and any biochemical marker, such as galactose-1-phosphate or residual GALT activity (not shown) were found.

**Table 3:** Correlation of ERT and RVP with other patient characteristics

ERT	IQ	EduS	Age	G1P
% correct	0.887*	0.705	−0.477	
% correct (happiness)			0.667	
% correct (sadness)	0.760*	0.587		−0.475
% correct (surprise)	0.646	0.616		−0.446
% correct (anger)	0.627	0.423		
% correct (disgust)	0.778*	0.599		−0.451
% correct (fear)				
Mean latency				
RVP				
A’ score	0.673	0.628		
Probability of hit	0.600	0.576		
Total correct rejections	0.726	0.645		
Total hits	0.600	0.576		
Probability of false alarm	−0.527		0.576	

Correlation coefficients of ERT and RVP measures with intelligence quotient (IQ), patients (professional) educational level (EduS), age, and galactose-1-phosphate (G1P). Only *à priori* significant correlations (*p* < 0.05) are shown. Where no such correlation exists, the cell was left blank. Only three correlations, marked with an asterisk (*), also have a significant *p*-value-FDR (< 0.05), which accounts for multiple comparisons (method of Benjamini & Yekutieli)

## Discussion

In this study, we aimed at deepening the neuropsychological phenotype of classical galactosemia patients by administering a series of tasks from the Cambridge Neuropsychological Test Automated Battery (CANTAB) to a Swiss cohort of 22 adult CG patients. This cohort represents 58% from totally 38 known patients in Switzerland.

In our cohort, we found the most robust deficits in facial emotion recognition (ERT) and rapid visual information processing (RVP). Most of the ERT and RVP outcome measures were correlated to overall IQ and education of the patients, much less to age. Apart from three instances (see Table 3), these correlations were no longer significant when the *p*-value was adjusted for multiple comparisons. Bad performance could not be explained by comprehension problems of the subjects with low IQ, since the recognition of ‘happiness’ was not different between patients and controls. Comparison of overall IQ and ERT performance in a scatter plot is shown in Additional file 3: Figure S2.

The patients performed also worse on several other outcome measures of the four other tasks, MOT, PAL, SSP and RTI. Probably due to the relatively small number of participants, only the Spatial Span (SSP) remained significant, when nonparametric testing and for multiple testing adjusted *p*-values were used (see Table 2).

To our best knowledge, this study is the first to examine facial emotion recognition in CG patients using the ERT of the CANTAB. Our results show that CG patients were able to correctly identify the basic emotions happiness and sadness but performed significantly worse on the more complex emotions anger, fear, disgust and surprise. Previous studies reported that CG patients have problems with social interactions and that they exhibit internalizing symptoms such as depression and anxiety [12, 17, 25]. In other studies, children with CG were also described as shy and withdrawn in social relationships [8, 26]. Interestingly, there seems to be a gap between the parents view and the patients’ self-perception of their emotional state: while the parents report considerable psychosocial difficulties, the patients themselves often fail to recognize them [12, 27]. Data concerning the patients’ behaviour and emotional state was mostly collected by means of questionnaires filled out by parents, teachers or patients themselves, but so far, little is known about the neuropsychological basis of the psychosocial impairment. Our results suggest that patients with CG have difficulties reading the facial expressions of their opposite correctly and therefore may not always react appropriately. Considering that the ERT is based on photographs of people acting the target emotions, it cannot be excluded that social conventions concerning these emotions play a role in the difficulties of the patients to recognize them. It is possible that CG patients also have difficulties to express their emotions or even to perceive and identify their own emotions. This could explain the differing opinions of parents and patients mentioned above. Importantly, these emotion recognition deficits may also be related to the observation that many galactosemia patients manifest autistic traits, but generally without fulfilling the diagnostic criteria.

Deficits in emotion recognition have also been described in other inborn errors of metabolism, such as Wilson’s disease [28] and tyrosinemia type I [29]. As in both studies different tests were used, conclusions by comparing the results can only be drawn cautiously. In the study with patients with Wilson’s disease, the most significant deficit was found in recognizing “anger”, while in our galactosemia patients, the most important difficult emotion was “fear”. The authors argue that patients with Wilson’s disease tend to react more aggressively to ambiguous social situations than healthy controls [28]. In contrast, galactosemia patients, as mentioned before, tend to be shy and withdrawn. In tyrosinemia type I patients, in turn, Van Ginkel and colleagues found less specific and less pronounced deficits in emotion recognition [29]. Nevertheless, similar to our findings, these were not completely explained by the correlation with IQ.

The RVP task revealed another weakness of CG patients. Our results showed that rapid visual information processing, a measure of sustained attention, is impaired in adult CG patients. Widhalm et al. postulated that galactosemic patients suffer from cognitive slowing and evaluated this outcome by means of reaction time tasks [30]. In their study, children with CG showed reduced ability to sustain visual attention, as well as attention deficits in central processing stages indicating a reduced processing capacity. Additionally, they had a remarkable impairment of information processing speed [30]. The significantly longer mean latency of patients in our study also suggests reduced velocity of visual information processing. In a study from Taiwan, RVP was administered to a relatively large cohort of adolescents with autism spectrum disorders (ASD) [31]. Compared to healthy controls, they performed significantly worse, even after adjusting for full IQ. The authors propose that RVP could serve as a trait marker for ASD. These findings are interesting in two respects: first, RVP abnormality in galactosemia patients may be another link to autism as discussed above for the ERT and second, this measure appears to be independent of IQ, at least in the normal IQ range. To our knowledge, no study has systematically investigated the correlation of RVP performance with low full IQ.

In the study of Widhalm et al. the CG children also performed significantly slower than controls on a task of simple reaction time [30]. Our patients however performed equally well in both measures of the simple-choice part and the reaction time of the five-choice part of the RTI. In contrast, the movement time in the five-choice part was slower, although this difference only reached significance when means were compared. This may be due to poor visual-motor integration described by previous studies [10, 32]. In addition, motor difficulties alone may have an influence, as it is easier to learn a uniform movement than an unpredictable one.

Results on PAL revealed problems with visual memory, which is most likely due to impaired visual perception as described by other researchers before [7, 15]. Reduced working memory capacity seems to be involved, too, as indicated by a significantly shorter span length of the CG patients compared to the controls on the SSP task. These findings are in line with previous studies reporting working memory scores of CG patients in the low average range [12, 13]. Furthermore, a recent study performed resting-state functional MRI on CG patients in order to assess the organization of core processing systems of the brain [33]. They found abnormalities in networks linked to spatial orientation, attention, sensory-motor integration and motor planning. In addition, altered connectivity was found in networks involved in visuospatial capacity and working memory. The alterations correlated with some neurocognitive tests which indicates a relation with the clinical phenotype [33].

## Conclusions

In conclusion, our study showed that CG patients have impaired visual perception, sustained visual information processing and visual-motor integration, thus confirming findings of previous studies. More interestingly, however, our study showed a deficit of facial emotion recognition in CG patients. To our best knowledge, this is the first time that this specific impairment has been demonstrated in the context of CG. The difficulty to recognise emotions correctly may have a considerable impact on patients’ social life. The selected CANTAB tasks proved useful to detect specific deficits of CG patients. Especially the ERT and the RVP appeared to be important for group discrimination. They could therefore be used in future studies such as functional MRI studies aiming to find neuronal correlates of the cognitive long-term complications, as well as surrogate markers of efficacy for potential new treatments. Finally, the findings of this study could also help to design programs for galactosemia patients aiming at the development of effective strategies to cope with the everyday consequences of these specific deficits in emotion recognition, in visual information processing and sustained attention.

## Methods

### Subjects and controls

The current study was approved by the local Ethics Committee and all subjects gave informed consent. The International Galactosemia Registry of the European Galactosemia Network (EGN Registry) was implemented in Switzerland in 2015, aiming at the inclusion of all CG patients, most of whom have been diagnosed by new-born screening since the mid-1960ies. All 38 known patients with CG in Switzerland who are ≥16 years of age, were contacted and invited to participate in this study. CG was defined by a known genotype of classical galactosemia or a residual GALT enzyme activity below 10%. Some patients declined to participate in the study because they did not feel well enough (*n* = 5). One of these suffered from a second condition (Down’s syndrome) and three were born before newborn screening. Others declined because they were not available, mainly for professional reasons (n = 5). For the remaining, the reason is not known (*n* = 6). The mean age of the non-included patients was 35.3 years (SD 13.1; range 18–59). The final sample consisted of 22 CG patients (59% females). Fifteen controls also completed the CANTAB test battery. They were recruited from a laboratory, administrative and medical staff. It was taken care that their level of education was similar to the level of education of the patient’s parents and male-female proportion was close or identical to the patient cohort, in order to reduce selection bias. In addition, three subjects with mild “Duarte” galactosemia were enrolled. They underwent identical testing as the patient group but were analysed separately. Due to their neuropsychological deficits, patients with CG often achieve lower education than their parents and non-affected siblings. In order to get a measure of the cognitive and psychosocial functioning of the patient families and the controls, of which no full IQ scores were available, we assessed and classified their level of education, as well as of the patients, as follows: “School without qualification”, i.e. either regular schooling not completed or special needs schooling. “School with qualification”, i.e. completed regular schooling and some additional professional training. “Vocational”, i.e. full professional education after obligatory school at age 16 and parallel to high school and college (this is the main educational path in Switzerland, with a good professional standing). “Undergraduate” and “Postgraduate” are the two University levels of the European Bologna system.

### Cognitive assessment

The study was part of a larger study conducted at the University Hospital of Bern in Switzerland, which included a full IQ assessment using the Wechsler Adult Intelligence Scale, Fourth Edition (WAIS-IV). Six tasks from the Cambridge Automated Neuropsychological Test Battery (CANTAB) were administered to all subjects including the controls in a session of approximately 60 min using German and French translations of the standardised test instructions and a Windows Surface touch-screen tablet. Thus, each task was explained to the subjects in a thorough and standardized way, and it was made sure that he/she had understood the instructions.

The following tasks were selected (see Additional file 1: Table S2 for a description of the tests):

1. *Motor Screening Task (MOT)*
2. *Paired Associates Learning (PAL)*
3. *Spatial Span (SSP)*
4. *Reaction Time (RTI)*
5. *Rapid Visual Information Processing (RVP)*
6. *Emotion Recognition Task (ERT)*

For reference, see http://www.cambridgecognition.com/cantab/cognitive-tests/ (Cambridge Cognition Ltd., 2017).

### Statistical analysis

Baseline characteristics were presented in a descriptive format, showing mean with standard deviation and median with range for age. The Student’s t-test was applied for mean, the Wilcoxon rank-sum test for median comparison between patients and controls. Frequencies with percentages were shown for categorical variables and the Chi-Squared test was used for comparison. The one sample t-tests was used to compare z-scores of controls to CANTAB normative data. For the comparison of the outcomes of the CANTAB sub-tests we again used the t-test for mean and the Wilcoxon rank-sum test for median, as well as linear models to compute *p*-values corrected for the influence of age, gender and the maximal educations of parents. As multiple statistical tests were performed, these *p*-values were adjusted using the method of Benjamini and Yekutieli to reduce the false discovery rate. The relative importance of outcome measures for predicting CG status was assessed by a random forest model consisting of 5000 trees, implemented in the randomForest R package. The random forest bootstrap estimate of error rate was 35.14%. The Spearman correlation was used and p-values were computed using Spearman’s rho statistics. All analyses were performed in version 3.4.1 of the R statistical environment.

## Additional files

Additional file 1: **Table S1:** Comparison of controls to CANTAB normative data. **Table S2:** CANTAB Tasks selected for the study. (DOCX 28 kb)

Additional file 2: **Figure S1.** Importance of individual CANTAB measures to group discrimination. The measures were ranked according to the relative importance of their contribution to distinguish between patients and controls. The higher the MDA (mean decrease in accuracy) the more important. Thus, the total number of emotions recognised correctly and the mean latency to respond are the two most important variables. The recognition of surprise (ERT-8) appears to be the emotion with the highest discriminative power among the six emotions tested, followed by fear (ERT-7). The full names of the outcome measures are listed in Table 2. (DOCX 61 kb)

Additional file 3: **Figure S2.** Scatter plot showing the correlation between overall IQ and ERT performance of the patients. Note that the subjects with the lowest IQ did not have the worst performance, suggesting that the latter was not due to a failure to understand the instructions. (DOCX 38 kb)

## Abbreviations

CANTAB: Cambridge Neuropsychological Test Automated Battery
CG: Classical galactosemia
ERT: Emotion Recognition Task
GALT: Galactose-1-phosphate uridyltransferase
IQ: Intelligence quotient
MOT: Motor Screening Task
PAL: Paired Associates Learning
RTI: Reaction Time
RVP: Rapid Visual Information Processing
SSP: Spatial Span
