# Supplementary material for: Deficits of facial emotion recognition and visual information processing in adult patients with classical galactosemia
Source: Orphanet J Rare Dis. 2019 Feb 26;14:56. doi: 10.1186/s13023-019-0999-3 (PMC6390315; doi:10.1186/s13023-019-0999-3)
Supplement: Supplementary file 1 — Table S1: Comparison of controls to CANTAB normative data. Table S2: CANTAB Tasks selected for the study. (DOCX 28 kb) [file 13023_2019_999_MOESM1_ESM.docx]

**Table S1: Comparison of controls to CANTAB normative data**

| **Description** | **Mean** | **StdDev** | **pValue** |
| --- | --- | --- | --- |
| MOT Mean latency | 0.581 | 0.998 | 0.414 |
| MOT Mean error | 1.118 | 0.837 | 0.011 |
| PAL First trial memory score | 0.025 | 0.709 | 1.000 |
| PAL Total errors (adjusted) | 0.213 | 0.380 | 0.425 |
| PAL Total errors (6 shapes, adjusted) | 0.160 | 0.467 | 1.000 |
| PAL Mean errors to success | 0.166 | 0.383 | 0.846 |
| PAL Mean trials to success | 0.224 | 0.353 | 0.338 |
| PAL Stages completed on first trial | -0.079 | 0.736 | 1.000 |
| PAL Total trials (adjusted) | 0.238 | 0.380 | 0.338 |
| SSP Span length | 0.710 | 0.934 | 0.217 |
| SSP Total errors | -0.354 | 0.948 | 0.988 |
| SSP Total usage errors | 0.028 | 0.620 | 1.000 |
| RTI Mean simple reaction time | 0.150 | 0.883 | 1.000 |
| RTI Mean simple movement time | 0.743 | 1.058 | 0.270 |
| RTI Mean five-choice reaction time | 0.378 | 0.956 | 0.981 |
| RTI Mean five-choice movement time | 0.970 | 0.885 | 0.033 |
| RVP A' score | 0.355 | 0.918 | 0.981 |
| RVP Probability of hit | 0.328 | 0.943 | 1.000 |
| RVP Mean latency | 0.803 | 0.959 | 0.159 |
| RVP Probability of false alarm | 0.118 | 0.818 | 1.000 |
| RVP Total correct rejections | 0.470 | 0.861 | 0.431 |
| RVP Total hits | 0.318 | 0.946 | 1.000 |

One sample t-tests of Z-scores. An adjusted *p*-value (FDR) was used to account for multiple comparisons (see Methods). No CANTAB normative data exist for the ERT.

**Table S2: CANTAB Tasks selected for the study.**

| Domain | Task | Task description | Outcome measures | Direction |
| --- | --- | --- | --- | --- |
| Introduction and screening for visual, motor or comprehension difficulties | Motor Screening Task (MOT) | A flashing cross which is shown in different locations on the screen has to be touched as fast as possible. | Mean latency (speed)  Mean error (accuracy) | Lower is better  Lower is better |
| Visual episodic memory and learning | Paired Associates Learning (PAL) | Boxes displayed on the screen are opened in a random order. Some of the boxes contain a pattern. Afterwards the revealed patterns are displayed in the middle of the screen, one at a time, and the participant has to allocate the pattern to the box where it was originally shown. In case of an error the boxes are re-opened and the patterns can be memorized again. Each stage can be attempted up to 10 times. Difficulty increases through the test as the number of patterns rises from one to eight. | First trial memory score (total number of patterns correctly located after first trial across all completed stages)  Total errors/trials (adjusted for stages completed)  Mean errors/trials to success | Higher is better  Lower is better  Lower is better |
| Working memory capacity | Spatial Span (SSP) | White boxes are displayed on the screen. Some of them briefly change colour in a varying sequence. The participant has to memorize the sequence and then touch the boxes which changed colour in the same order as they were displayed before. The sequence elongates at each level from initially two boxes to nine in the end. The participant has three attempts at each level. | Spatial span (longest sequence successfully recalled)  Total errors | Higher is better  Lower is better |
| Mental and motor response speed | Reaction Time (RTI) | A yellow dot appears on the screen, in either a single location (simple) or in one of five possible locations (five-choice). The participant has to hold down the button on the press pad until the dot appears. As soon as he perceives the dot the participant has to release the button and touch the yellow dot on the tablet as fast as possible. | Reaction time (simple or five-choice)  Movement time (simple or five-choice) | Lower is better  Lower is better |

| Visual sustained attention | Rapid Visual Information Processing (RVP) | A box is displayed in the middle of the screen in which digits from 2-9 are presented in a pseudorandom order at a rate of 100 digits per minute. Participants have to identify three-digit target sequences and respond by pressing the button on the press pad. | A’ score (measures how good the subject is at detecting target sequence using total hits and total false alarms)  Probability of hit  Probability of false alarm  Mean latency | Higher is better  Higher is better  Lower is better  Lower is better |
| --- | --- | --- | --- | --- |
| Ability to identify emotions in facial expressions | Emotion Recognition Task (ERT) | Computer converted images containing facial features of real individuals each showing a specific emotion are displayed on the screen. One image is only shown for 200ms and the participant must then choose the emotion expressed in the image out of six basic emotions. These emotions are happiness, sadness, anger, fear, disgust and surprise. | Percent correct (for each emotion)  Mean latency | Higher is better  Lower is better |
