# Supplementary material for: Deficits of facial emotion recognition and visual information processing in adult patients with classical galactosemia
Source: Orphanet J Rare Dis. 2019 Feb 26;14:56. doi: 10.1186/s13023-019-0999-3 (PMC6390315; doi:10.1186/s13023-019-0999-3)
Supplement: Supplementary file 2 — Figure S1. Importance of individual CANTAB measures to group discrimination. The measures were ranked according to the relative importance of their contribution to distinguish between patients and controls. The higher the MDA (mean decrease in accuracy) the more important. Thus, the total number of emotions recognised correctly and the mean latency to respond are the two most important variables. The recognition of surprise (ERT-8) appears to be the emotion with the highest discriminative power among the six emotions tested, followed by fear (ERT-7). The full names of the outcome measures are listed in Table 2. (DOCX 61 kb) [file 13023_2019_999_MOESM2_ESM.docx]

**Figure S1**

SSP-2 (relative errors)

SSP-2 (relative e)

SSP-3 (relative usage errors)

ERT-3 (happiness)

RVP-4 (probability false alarm)

PAL-1 (memory score)

RTI-4 (5-choice movement time)

RTI-2 (simple movement time)

PAL-6 (stages compl 1st trial)

MOT-2 (mean error)

ERT-4 (sadness)

PAL-3 (tot errors 6 shapes)

RTI-3 (5-choice reaction)

ERT-6 (disgust)

PAL-5 (mean trials to succ)

PAL-7 (tot trials adjusted)

PAL-4 (mean errors to succ)

PAL-2 (tot errors adjusted)

ERT-2 (% correct)

SSP-1 (span length)

MOT-1 (mean latency)

RVP-3 (mean latency)

RVP-2 (probability of hit)

ERT-5 (anger)

RVP-6 (tot hits)

RVP-1 (A’ score)

RVP-5 (tot correct rejections)

ERT-7 (fear)

ERT-8 (surprise)

ERT-9 (mean latency)

ERT-1 (total correct)

0

10

20

30

40

MDA
