# Supplementary figures and images for: Deficits of facial emotion recognition and visual information processing in adult patients with classical galactosemia
Source: Orphanet J Rare Dis. 2019 Feb 26;14:56. doi: 10.1186/s13023-019-0999-3 (PMC6390315; doi:10.1186/s13023-019-0999-3)

**Figure S2**


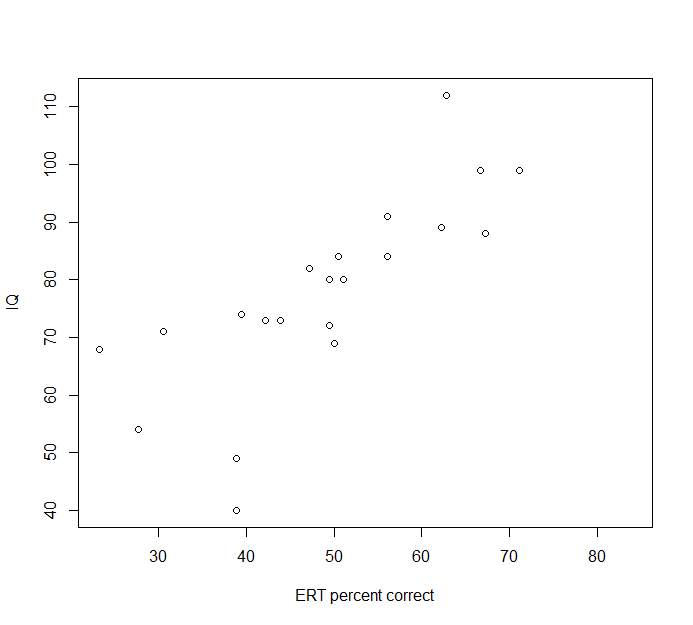

Supplement: Supplementary file 3 — Figure S2. Scatter plot showing the correlation between overall IQ and ERT performance of the patients. Note that the subjects with the lowest IQ did not have the worst performance, suggesting that the latter was not due to a failure to understand the instructions. (DOCX 38 kb) [file 13023_2019_999_MOESM3_ESM.docx]
